# Fracture Assessment of PEEK under Static Loading by Means of the Local Strain Energy Density

**DOI:** 10.3390/ma10121423

**Published:** 2017-12-13

**Authors:** Mirco Peron, Seyed Mohammad Javad Razavi, Jan Torgersen, Filippo Berto

**Affiliations:** Department of Industrial and Mechanical Engineering, Norwegian University of Science and Technology, Richard Birkelands vei 2b, 7491 Trondheim, Norway; javad.razavi@ntnu.no (S.M.J.R.); jan.torgersen@ntnu.no (J.T.); filippo.berto@ntnu.no (F.B.)

**Keywords:** tensile behavior, PEEK, strain energy density, SED, notch

## Abstract

Polyetheretherketone (PEEK) has gained interest in many industrial applications due to its high strength-to-weight ratio, excellent heat tolerance and high corrosion resistance. Stress concentrators such as notches and geometrical discontinuities are present in many such components necessitating the reliable assessment of notch sensitivity of PEEK in monotonic tension. Here we evaluate the applicability of the strain energy density (SED) approach for the assessment of the fracture strength of experimentally tested notched geometries subject to corrosion. The fracture behavior of neat, circumferentially razor-grooved dog-bone specimens and circumferentially U-notched specimens with different notch radii can be predicted with a discrepancy lower than ±10%. Reliable predictions are shown on two previously published datasets employing both computed and published mechanical properties as inputs for the SED calculations. This report presents the first successful application of SED for PEEK as well as the successful prediction of tensile behavior in corrosive environments. This opens the road towards future applications of PEEK in fields its compliant use is of growing popularity.

## 1. Introduction

For applications where high specific mechanical properties and high corrosion resistance are needed, advanced polymers are attractive [1]. Polyetheretherketone (PEEK) emerged as a successful engineering polymer due to its high strength-to-weight ratio, excellent tribocorrosion and thermal properties (its glass transition temperature is of 143 °C [2]). It is a reliable substitute for metallic materials in many industrial applications and a key material in food processing, for impeller wheels in regenerative pumps, in high pressure and temperature pipes and hose couplings as well as gearwheels [3,4].

Numerous authors have reported on the strain rate dependency of this material. El-Qoubaa and Othman [5] assessed the tensile mechanical behavior of PEEK at room temperature at a range of strain rates (from 0.001 to 1000 s^−1^). The yield stress increased with strain rate, with the strain rate sensitivity reported higher at higher rates. Albérola et al. [6] reported an increase of Young’s modulus and yield stress by increasing the strain rate from 10 to 200 s^−1^. Similarly, strain rate sensitivity was weaker for strain rates 0.00001 to 10 s^−1^. Most industrial components contain geometrical discontinuities (notches) negatively affecting fracture and fatigue strength [7,8,9]. Despite their importance for dimensioning against failure, a lack of knowledge exists for assessing the notch sensitivity of PEEK. To the best of authors’ knowledge, only Sobieraj et al. [10] and Chen et al. [11] assessed notch effects on tensile strength of this semi-crystalline polymer. Sobieraj investigated the static behavior of un-notched and notched PEEK in corrosive environment at two different strain rates (0.1 and 0.5 s^−1^), whereas Chen explored the fracture behavior of four different axisymmetric notched specimens. Both pioneering studies state that PEEK is notch-sensitive; yet a fundamental and reliable failure criterion for PEEK’s tensile behavior needs to be found. This criterion should not only predict the tensile behavior independent from type of PEEK base material and geometry, but should also account for degradation effects due to environmental impacts. For the first time, we aim to provide such a criterion employing an energy-based approach, i.e., the strain energy density (SED) criterion. As years go by, from the introduction of the Absorbed Specific Fracture Energy (ASFE) for the determination of the fracture toughness and the J-integral of low and medium strength structural material [12], several energy-based approaches were developed. Among them, the strain energy density (SED) criterion has revealed to be robust for predicting tensile and fatigue behavior of various metals weakened by geometrical discontinuities [8,13,14,15,16]. In addition, the authors recently provided indications that suggest its applicability for polymeric materials [17]. Polyurethane foams were tested under different loading conditions providing a satisfactory prediction of experimental tensile strength (+5% to −10% relative errors) [18]. Furthermore, polymethylmethacrylate’s (PMMA’s) fracture behavior under different loading conditions and temperatures were assessed using SED [19,20]. The authors tested specimens at room temperature and at −60 °C. For both temperature conditions, the SED criterion provided reliable predictions. The entire spectrum of 70 experimental tensile strength values fell within a scatterband of ±15%. Most importantly, the majority of values fell inside the range of ±10% scatter band, which is considered a reasonable prediction in the engineering field according to a recent review dealing with brittle and quasi-brittle fracture of various materials [7]. Here, we show the reliable prediction of the fracture strength of PEEK comparing theoretical SED results to the experimental data published by Sobieraj et al. [10] and Chen et al. [11]. In what follows, the reader will be presented with a reliable estimation of the characteristic strain rate sensitivity that is independent from specimen geometry, from the reference dataset and that accounts for the impact of the degrading environment surrounding the specimen.

## 2. Experimental Reference Data

**Figure 1:**
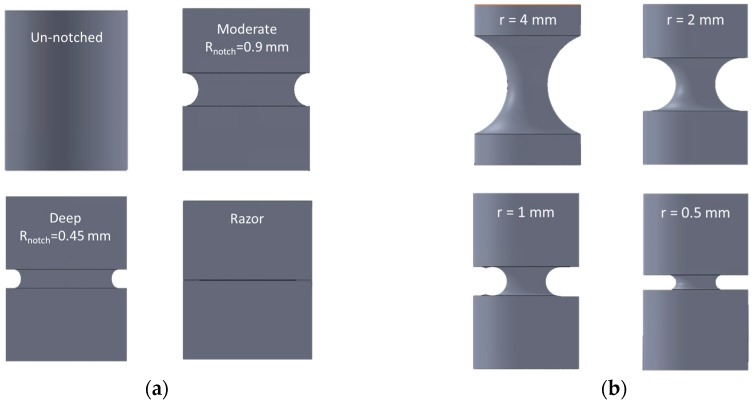
Schematic view of specimen geometries: (**a**) Geometries tested in Ref. [10]; (**b**) Geometries tested in Ref. [11].

Sobieraj et al. [10] examined the stress-strain behavior of neat and notched PEEK under uniaxial loads. Their notched specimens are circumferentially grooved round bars with an 8 mm outer diameter weakened by three different types of notches. These are circumferentially U-notched geometries with a 6 mm inner diameter with moderate (0.9 mm) and deep (0.45 mm) notch radii. In addition, a circumferentially razor grooved dog-bone was investigated (Figure 1a).

**Figure 2:**
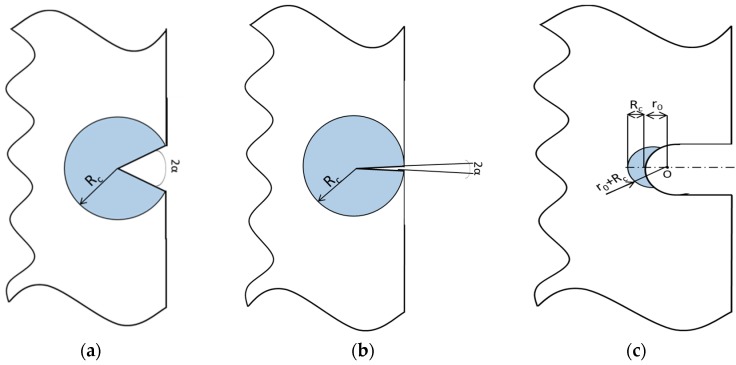
Control volume for sharp V-notch (**a**), crack case (**b**) and U notch (**c**) under mode I loading.

The specimens, made by OPTIMA LT1™ (Invibio, Inc., West Conshohocken, PA, USA), were tested in a corrosive environment with two different strain rates at 0.1 and 0.5 s^−1^, respectively. The maximum axial true stresses together with the experimental scatter are listed in Table 1. The strain rate dependence of Young’s modulus and yield stress was found to be weak (Figure 2), confirming the results obtained in reference [6] in which strain rates from 0.00001 s^−1^ to 10 s^−1^ were reported to negligibly affect these properties.

Chen et al. [11] assessed the fracture behavior of PEEK 450G (Röchling Group, Mannheim, Germany) testing axisymmetric specimens machined with notched radii of 0.5, 1, 2 and 4 mm (Figure 1b), respectively. The specimens were manufactured with an outer diameter of 8 mm and a net section of φ = 3 mm. The tensile tests were carried out at room temperature under a strain rate of 0.1 s^−1^ and the true stresses are listed in Table 2 (no experimental scatter were reported in the reference text).

## 3. Brief Introduction of the SED Approach

The SED criterion states that the failure of a component occurs when the total strain energy, $\overset{-}{W}$, averaged in a circular control volume of radius *R_c_* surrounding a crack or notch tip, reaches its critical value *W_c_* [21]. This is material dependent [7]. SED was applied with excellent results to the assessment of the tensile and fatigue behavior of different materials weakened by several notch geometries [22,23,24]. Critical parameters can be analytically derived with only few material properties [21]: The ultimate tensile strength of the un-notched material *σ_t_*, the Young’s modulus E and the fracture toughness, labeled as *K_IC_* in accordance with the convention adopted in [25]. In the case of ductile material, the ultimate tensile strength should be replaced with the maximum true stress or a fictitious stress determined using the so-called Equivalent Material Concept [22]. In agreement with Beltrami [26], the critical value of the total strain energy can be determined by the following:

(1) $$W_{c}=\frac{\sigma_{t}^{2}}{2E}$$

In plane problems the control volume becomes a circular sector or a circle, for V-notches or cracks respectively (Figure 2a,b). The critical radius *R_c_* is defined as follow [21]:

(2) $$R_{c}=\frac{\left(1+v\right)\left(5-8v\right)}{4\pi }{\left(\frac{K_{Ic}}{\sigma_{t}}\right)}^{2}$$

where *ν* is the Poisson’s ratio of the material. For a blunt V-notch or a U-notch (Figure 2c), the volume is assumed to be of a crescent shape, where *R_c_* is the depth measured along the bisector line. The outer radius of the crescent shape is equal to *R_c_* + *r*_0_ with *r*_0_ being the distance between the notch tip and the origin of the local coordinate system (Figure 2c). Such a distance depends on the notch-opening angle 2*α* and the notch root radius *ρ*, according to the expression:

(3) $$r_{0}=\rho \frac{\left(\pi -2\alpha \right)}{\left(2\pi -2\alpha \right)}$$

The accuracy of this analytically obtained critical radius has been validated in previous works by Lazzarin and co-authors [7,8].

However, the fracture toughness is not always available due to the difficulties and time consuming calculations. Yet, an estimate of the critical radius can be obtained as the radius at which the critical SED values for two different specimen geometries are identical [27]. The computation of this SED value can be performed utilizing finite element (FE) modeling. In this work, we employ the Ansys^®^ (Canonsburg, PA, USA) drastically reducing the effort that otherwise would have been spent using the complex theoretical derivation obtained in reference [21].

## 4. Finite Element Model

**Figure 3:**
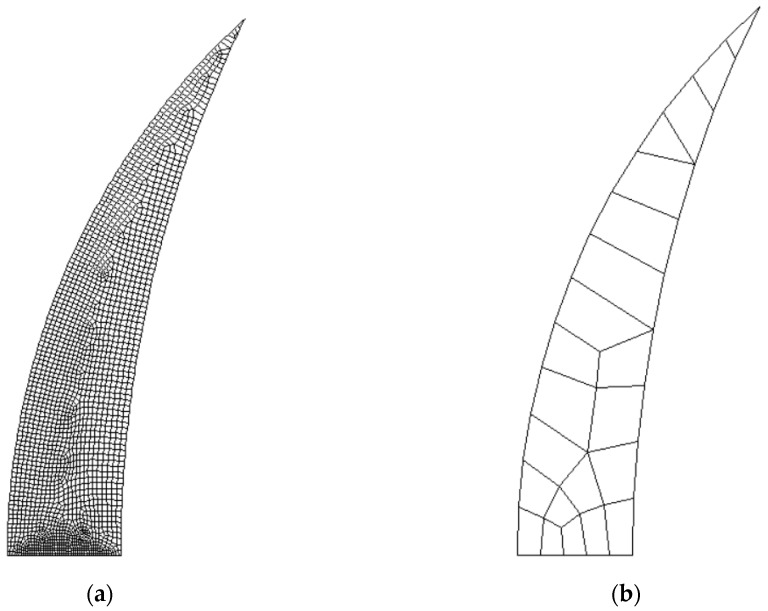
Evaluation of the strain energy density (SED) mesh sensitivity: (**a**) Number of elements = 2049, $\overset{-}{W}$ = 2.8431 MJ/m^3^; (**b**) Number of elements = 23, $\overset{-}{W}$ = 2.8398 MJ/m^3^.

In order to obtain the SED value, axisymmetric linear elastic 2D analyses were performed on the notched models. Due to the double symmetry of the geometry, only one quarter of the specimens were modeled. The 8-nodes axisymmetric element plane 83 was selected for these analyses. A mesh convergence study was undertaken to ensure that a proper number of elements was used in FE modelling, with elements size at the crack tip ranging from about 10^−3^ mm to 10^−1^ mm. The results are independent from the mesh being the difference is only 0.11% between the SED value for a coarse mesh and that for a fine mesh (Figure 3), and thus a coarse mesh was adopted for the analyses. The mesh-insensitivity of the SED approach was previously reported also by Berto and Lazzarin [23] for cracked and notched specimens. This represents one of the main advantages of this approach, together with the capability of assessing the tensile behavior of different materials regardless of the geometry. In fact, FE codes could also be used for determining the tensile strength of different components with the stress-strain curve of the un-notched material as the only input. In this case, however, the results are extremely dependent on the mesh, thus requiring high computational efforts and time for achieving reliable results. We hence aim to provide a solution to this challenge that is independent from the mesh and easy to compute.

**Figure 4:**
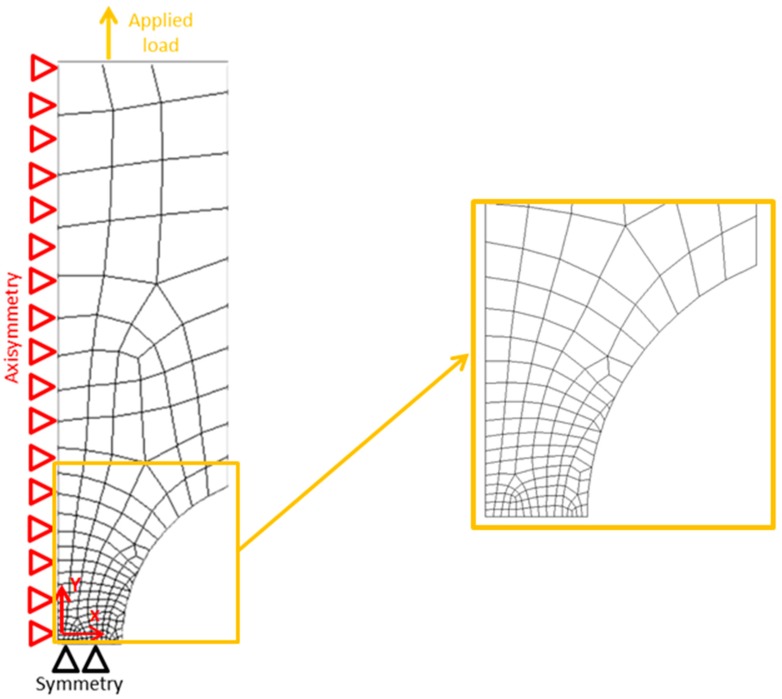
Typical mesh pattern of the finite element model near the notch tip and schematics of boundary conditions.

Figure 4 illustrates the mesh pattern and the boundary conditions used for FE analyses. Symmetric boundary conditions were used for vertical and horizontal symmetry lines of the models; however, the top side of the model was able to move along the loading axis.

## 5. Results and Discussion

### 5.1. Prediction with Known Critical SED

**Figure 5:**
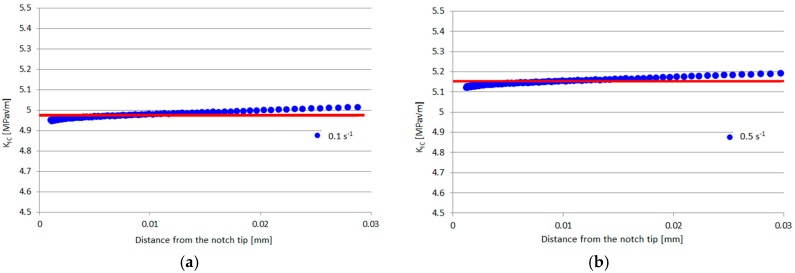
Estimation of the fracture toughness for a strain rate of 0.1 s^−1^ (**a**) and 0.5 s^−1^ (**b**).

Sobieraj et al. tested un-notched and notched PEEK specimens under different strain rates and in a corrosive environment, and in this section their results were analyzed in terms of SED. The application of the SED approach requires the critical value of the radius *R_c_* of the control volume and that of the strain energy density *W_c_*. The critical SED value can be simply evaluated using Equation (1), leading to a critical SED value of 7.278 and 6.38 MJ/m^3^ under a strain rate of 0.5 and 0.1 s^−1^, respectively. Concerning the control volume; in reference [10], the fracture toughness has not been reported. Determining the stress distribution at the crack tip by means of numerical simulations, the fracture toughness can be estimated via the Notch Stress Intensity Factors (NSIFs) formulation derived by Gross and Mendelson [28]:

(4) $$K_{I}=\sqrt{2\pi }\underset{r\to 0}{\lim}r^{\left(1-\lambda_{I}\right)}\left[\sigma_{\theta \theta }(r,\theta =0)\right]$$

where *r* and *θ* is the radial and angular coordinate of a polar coordinate system centered at the notch tip, *σ_θθ_* is the stress component according to the coordinate system and *λ_I_* is the William’s eigenvalue [29]. Applying the tensile strength obtained experimentally from the razor specimen, for which *λ_I_* is equal to 0.5, the fracture toughness was estimated as 4.99 and 5.16 MPa√m for a strain rate of 0.1 and 0.5 s^−1^, respectively (Figure 5). The increment of the fracture toughness increasing the strain rate was also observed in reference [30].

In the FE code, the material was assumed isotropic and linear elastic with the Young’s modulus *E* = 3500 MPa and the Poisson’s ratio *ν* = 0.36 as in [10]. The 8-nodes axisymmetric element plane 83 was selected for these analyses with elements size at the crack tip of about 10^−4^ mm. Using these values in Equation (2), the critical radius results to be 0.128 and 0.12 mm for specimens tested at 0.1 and 0.5 s^−1^, respectively. The small difference in radius is due to the material’s limited strain rates sensitivity below 10 s^−1^ [6]. We predicted the tensile strength by means of FE modeling. In the predictions, the critical failure load is taken as the one rendering an SED value of the three different specimen geometries under study (moderate, deep and razor), which is equal to the critical one obtained with Equation (1). The SED value is proportional to the square of the applied stress. A unit load was applied in the simulations. The predicted tensile strength was then computed as the square root of the ratio between the critical strain energy density and the SED value. The SED prediction of the tensile failure for both the strain rates are reported in Table 3.

For the geometries and test conditions reported in [10], the presented approach provides suitable prediction of tensile failure, where deviations (the difference between the experimental and predicted values) are generally lower than 4%, for predictions of moderate and razor grooved merely even below 1%. Impacts of the corrosive test environment were successfully accounted for. Yet PEEK is available in different grades and formats depending on the application and processing method. The question remains, if the presented approach still holds for different PEEK grades and geometries, especially when the critical SED value cannot be analytically obtained but the fracture toughness is known [31,32,33]. This will be the topic of the next section.

### 5.2. Predictions with Known Fracture Toughness

**Figure 6:**
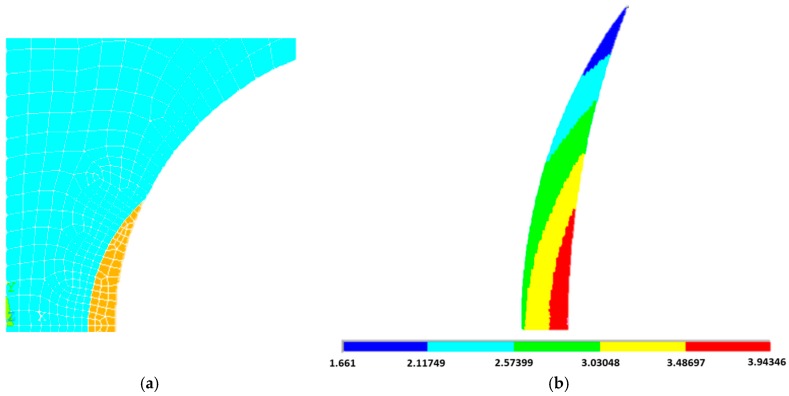
Control volume (yellow) in the axisymmetric specimen machined with a notch radius of 4 mm (**a**) and contour plot of the SED (in MJ/mm^3^) distribution within the control volume in the specimen weakened by a notch radius of 4 mm (**b**).

Chen et al. [11] assessed the tensile behavior of PEEK specimens weakened by four different notched geometries. The analytical formulation for determining the SED critical value is not applicable as the authors did not test un-notched reference specimens. As previously described, it is possible to evaluate the critical radius and SED value utilizing FE code only. We may change the radius of the control volume of the specimens with two different control radii and iteratively compute the SED value until a satisfying convergence is reached. This approach was shown in the previous section, however, for the type of material studied here, the fracture toughness can be taken from literature [34]. In this way, Equation (2) gives us 0.37 mm critical radius leading to a SED value of 2.84 MJ/m^3^ for axisymmetric specimens with 4 mm notch radius (obtained with Ansys^®^, Figure 6).

Assuming this material is isotropic, linear elastic FE analyses with an elastic modulus of 4000 MPa and Poisson’s ratio of 0.38 were conducted [11]. The difference in the elastic properties from those reported in the previous section is due to different grade of the material provided by two different material suppliers. Different grades correspond to different degree of crystallinity and to different length and conformations of macromolecular chains influencing the polymer’s mechanical properties. In fact, Starkweather et al. [35] reported lower mechanical properties (elastic modulus, yield stress and tensile strength) with a low degree of crystallinity. In addition, Perkins et al. [36] found an increase in the strain to break with higher molecular weight. Finally, the differences in grade of the material under study are reflected on critical radius and critical SED value, which are different from those obtained in the previous section (data from [10]). Grade related property discrepancies also exist for PMMA [21]. The results of the tensile strength prediction for the specimens weakened by a notch radii of 0.5, 1 and 2 mm, respectively, are listed in Table 4.

The deviation between the predicted values and the experimental data is in a compliant scatter range with reference to previous well-known studies in the engineering field.

**Figure 7:**
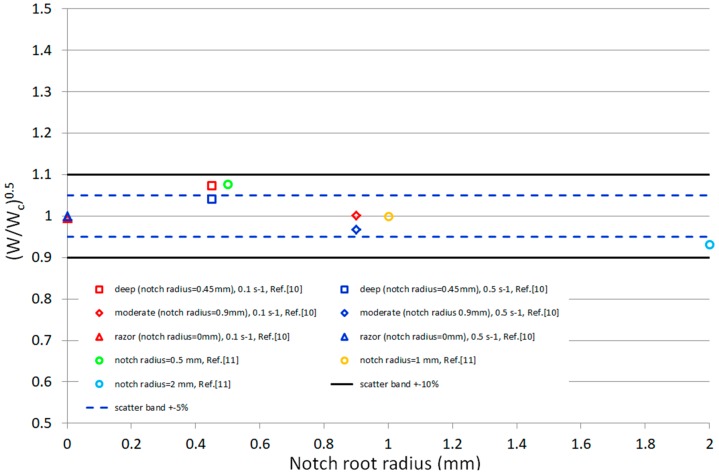
Synthesis of fracture data in terms of normalized SED. The data from reference [10] are labeled in the legend reporting the geometry followed by the strain rate, while the data from reference [11] report the notch radius in the legend.

To benchmark the choice of the considered control volume, we plotted the square root of dimensionless strain energy density (*W*/*W_c_*)^0.5^ as a function of the notch root radius (Figure 7). It appears proportional to the fracture loads of the tested notched samples; the control volume is hence appropriate with respect to various notch tip radii effects, environmental conditions and strain rates. The majority of fracture load predictions are well inside the scatter band of ±10% with a considerable number of predicted data within ±5%. This is well in the performance range with many studies of a SED database that appeared in a recent review dealing with brittle and quasi-brittle fracture of various materials [7]. We hypothesize the SED’s routine is applicabile for mechanical properties prediction of PEEK in various material grades and geometrical configurations with excellent predictive power.

## 6. Conclusions

The strain energy density (SED) approach was previously developed for the assessment of tensile and fatigue behavior of metals weakened by different notch geometries. For the first time, we successfully employed the SED to predict the tensile strength of PEEK in corrosive environments. Various notch geometries under different strain rates and environmental conditions were simulated and show well agreement with experimental results. Simulations both capture the strain- and notch-sensitivity of the material independent from its grade. Predictive performance of the tensile strength of PEEK are characterized by a discrepancy to the experimental data in the range of ±10%, the performance range considered acceptable in many studies of a SED database [7]. This was done independently of the type of PEEK material.

## Figures and Tables

**Table 1:** Tensile properties of notched and un-notched polyetheretherketone (PEEK) specimens under different strain rates.

Condition	Un-Notched		Moderate		Deep		Razor	
Strain rate (s^−1^)	0.1	0.5	0.1	0.5	0.1	0.5	0.1	0.5
Max axial true stress (MPa)	211 ± 8.16	225 ± 5.35	132 ± 1.11	135 ± 0.42	127 ± 2.33	129 ± 1.36	119 ± 4.92	123 ± 4.33

**Table 2:** Tensile properties of notched axisymmetric specimens.

***r*** **(mm)**	4	2	1	0.5
**Maximum axial true stress (MPa)**	160	145	150	177

**Table 3:** Prediction of tensile failure of moderate, deep and razor specimens using the SED approach.

Specimen Geometry	Strain Rate (s^−1^)	Experimental Data (MPa)	SED Prediction (Mpa)	Deviation
Deep	0.1	127	118	−6.8%
	0.5	129	124	−4%
Moderate	0.1	132	132	−0.1%
	0.5	135	139	+3.3%
Razor	0.1	119	118	−0.8%
	0.5	123	124	+0.8%

**Table 4:** Prediction of tensile failure of axisymmetric specimens machined with notched radii of 0.5, 1 and 2 mm using the SED approach.

Specimen Geometry	Experimental Data (MPa)	SED Prediction (MPa)	Deviation
*r* = 0.5	177	165	−6.8%
*r* = 1	150	155	+3.3%
*r* = 2	145	156	+7.5%
